# 

**Published:** 1902-02-15

**Authors:** 


### ANNOUNCEMENTS.

### CHICAGO ODONTOGRAPHIC SOCIETY.

At the annual meeting of the Odontographic Society, held Monday, December 16, 1901, the following officers were elected for the ensuing year: President, C. N. Johnson; vice-president, W. T. Reeves; secretary, Frank H. Zinn; Treasurer, George N. West; board of directors, George B. Perry, F. E. Roach, L. O. Green; board of censors, F. B. Noyes, W. Girling, D. A. Hare; program committee, C. E. Bentley, L. S. Tenney, H. J. Goslee. The fifteenth anniversary of the society will occur in December, 1902. It is the intention of the society to celebrate the event by giving a rousing "clinic," extending over two days, and a meeting that will be memorable in its history. Prominent members from all parts of the country are to be invited to be present. The program committee have already commenced plans for one of the most notable meetings that has been held in this country.

### OHIO STATE DENTAL SOCIETY.

At the thirty-sixth annual meeting of the Ohio State Dental Society, held at Columbus, December 3–5, 1901, the following officers were elected for 1902: President, Otto Arnold, Columbus; first vice-president, J. B. Beauman, Columbus; second vice-president, J. F. Stephan, Cleveland; secretary, S. D. Ruggles, Portsmouth; treasurer, C. I. Keely, Hamilton.

